# Monkeypox Presenting as a Hand Consult in the Emergency Department: Two Case Reports

**DOI:** 10.1177/15589447231177098

**Published:** 2023-06-08

**Authors:** David T. Mitchell, James A. Mentz, Yuewei Wu-Fienberg, Wendy Chen, Matthew R. Greives, Erik S. Marques

**Affiliations:** 1 The University of Texas Health Science Center at Houston, USA

**Keywords:** mpox, monkeypox, hand infection, monkeypox lesion, infection, diagnosis

## Abstract

The ongoing outbreak of the monkeypox virus (now referred to as “mpox”) was deemed a public health emergency by the World Health Organization in 2022. The United States now reports the highest number of mpox cases, with 29 980 cases and 21 deaths as of January 11, 2023. The most common presenting symptom is a pruritic, vesicular rash that commonly involves the hands. While covering hand call, our division has encountered 2 cases of mpox in the emergency department for which the chief complaint was a hand lesion. Because hand surgeons will be called upon to make an initial diagnosis, the purpose of these case reports is to describe the presentation, disease course, treatment, and outcomes of these mpox patients. These patients had both uncontrolled HIV as well as other sexually transmitted disease. Symptoms included painful vesicular hand lesions with ulceration and eventual central necrosis, followed by similar lesions on the face, trunk, and genital area. Diagnosis was made using nucleic acid amplification testing through polymerase chain reaction. The patients were treated with restoration of immunity through control of HIV as well as treatment of all secondary bacterial infections. One patient died in the hospital, and the other survived without any long-term defects.

## Introduction

Monkeypox virus (now referred to as “mpox” by the World Health Organization [WHO] to reduce stigma and issues associated with terminology) is a viral disease causing an ongoing outbreak that started in London, United Kingdom in May 2022.^1^ The WHO declared the outbreak a public health emergency of international concern in July 2022. The United States is now reporting the highest number of mpox cases in the world at 29 980 cases and 21 deaths as of January 11, 2023.^2,3^ The most common symptom is a rash on the hands, feet, chest, face, mouth, or genitals, which may begin as painful or pruritic macules and papules that progress to vesicles and pustules that umbilicate and scab before healing, with an incubation period of 3 to 17 days, during which a person may not have symptoms.^4^ Other common symptoms include fever, malaise, chills, headache, and swollen lymph nodes.^5^ The Centers for Disease Control and Prevention (CDC) reports that current cases comprise 99% men, 94% of whom have reported recent male-to-male sexual or close intimate contact.^6^ There have also been reports of coinfections with sexually transmitted diseases, such as syphilis, gonorrhea, chlamydia, and herpes.^7^

While covering hand call, our division encountered 2 cases of mpox in the emergency department (ED) for which the initial chief complaint was a hand lesion. With hand lesions as a potential presenting symptom for mpox, hand surgeons may be called upon to make an initial diagnosis. The purpose of this article is to describe our experience with the presentation, diagnosis, treatment, and disease course of mpox in hand patients to allow for earlier recognition and proper management for this unique patient population.

## Methods

In this article, the authors will conduct a retrospective chart review of the 2 cases of mpox that were presented to the ED as hand lesions. Clinical documentation and photographs are assembled to describe the presentation, diagnosis, disease course, treatment, and outcomes of patients with this disease. All health information has been deidentified.

## Results

### Patient A

#### Presentation

**Figure 1.**
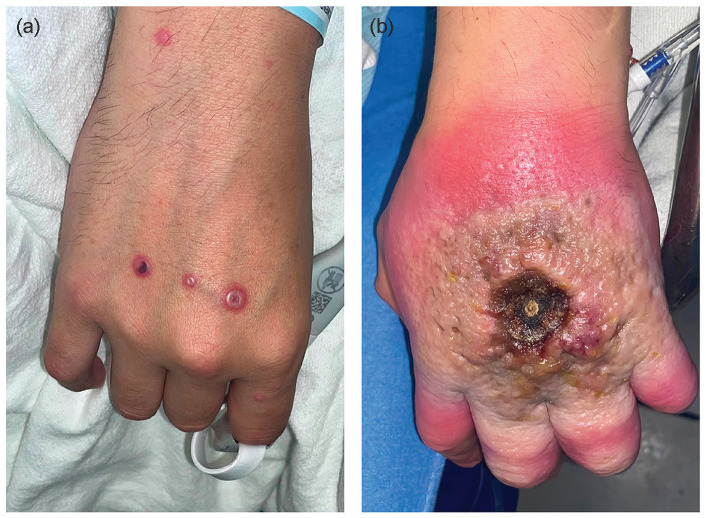
(a) Right-hand and (b) left-hand lesions of patient A upon presentation. The patient had similar umbilicated lesions on his entire body.

Patient A is a 41-year-old man with uncontrolled HIV, who presented to the ED with a 3-week history of worsening left-hand lesion (Figure 1b) that was soon followed by numerous scattered lesions on the trunk, face, right hand (Figure 1a), and perineal and scrotal skin. These lesions began as pruritic macules, and then, over the course of days progressed to raised macules that consolidated into vesicles. The vesicles, with clear fluid, became pustular with opaque fluid and umbilicated around days 5 to 7. The umbilicated pustules developed central necrosis over the next 2 weeks. On presentation, the first lesion, which was on his hand, had been centrally necrosed for a week. His pain was most severe in the left hand and perianal region. Review of symptoms was negative except for fever and rectal pain. On physical examination, the patient’s hand lesion was tender, blanching erythematous, and without expressible fluid. He had no functional deficits.

#### Diagnosis

Due to immediate suspicion for mpox, the infectious disease team was consulted. Hand x-ray, computed tomography, and magnetic resonance imaging confirmed the absence of osteomyelitis or fluid collections in the hand. Swab sample of the lesion was sent for polymerase chain reaction (PCR) nucleic acid amplification testing (NAAT) analysis, which confirmed mpox 2 days later. At presentation, his cluster of differentiation 4 (CD4) was 104, HIV viral load 2.2 million. He was also found to have cytomegalovirus viremia, syphilis, and herpes simplex virus of unknown duration. Blood cultures were negative.

#### Treatment

The goal of our treatment was to control the patient’s active infections while he reconstituted his immune system. The patient was started on an aggressive course of antibiotics for suspected superimposed cellulitis around his lesions and antivirals for his viral illnesses. He was started on bictegravir/emtricitabine/tenofovir alafenamide antiretroviral therapy for his HIV. For his mpox, the patient was started on a regimen of tecovirmat antiviral therapy. The patient would later receive 2 rounds of vaccinia immune globulin as well as weekly cidofovir injections. Wound care consisted of povidone-iodine paint to the scabbed lesions to prevent superinfection, silver sulfadiazine over eschars, and, eventually, twice-daily povidone-iodine soaks after the lesions coalesced.

#### Disease course/outcome

**Figure 2.**
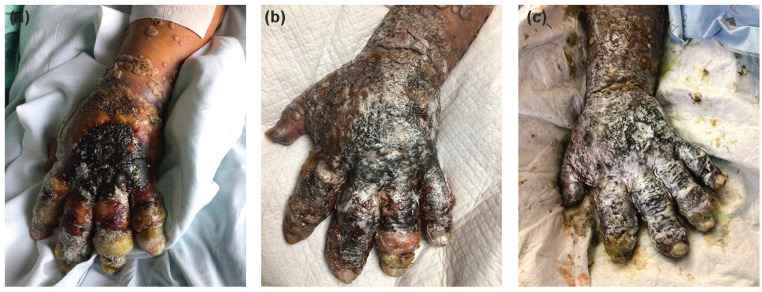
Progression of patient A’s hand lesions. Pictures are (a) 30 days, (b) 45 days, and (c) 60 days after initial presentation. He had similar lesions over his entire body that coalesced.

Patient A’s course was complicated by progression of his mpox proctocolitis to the point that it caused bowel obstruction and resulted in colonic dilatation and ischemia requiring colectomy. Control of his HIV took weeks and was complicated by immune reconstitution inflammatory syndrome, resulting in clinical deterioration. The patient developed multidrug-resistant urinary tract infection and pneumonia in the hospital, as well as a pleural effusion. The patient underwent thoracentesis and was found to have lymphoma. He developed acute kidney injury and was unable to continue his numerous nephrotoxic medications. At the end of his course, the patient’s lesions had coalesced to cover his chest, face, and hands, resulting in stiffness in all hand joints (Figure 2a-2c). Without any reasonable ability to treat his infection or malignancy, his family decided to deescalate care. The patient finally died of metabolic acidosis 2 months after presentation.

### Patient B

#### Presentation

**Figure 3.**
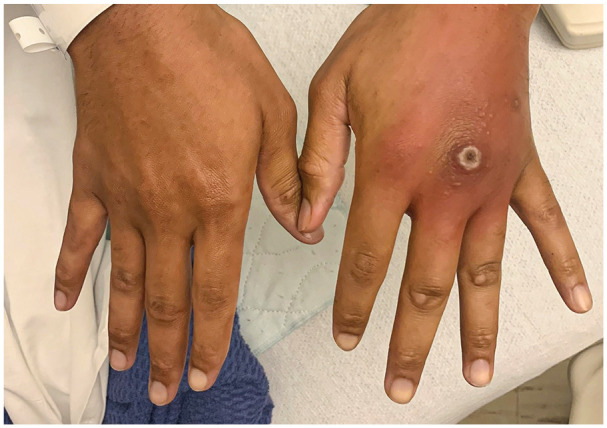
Bilateral hands of patient B upon presentation.

Patient B is a 29-year-old man with a medical history of HIV of 11 years, with poor medication compliance for the past 6 months, who presented to the ED with a 2-week history of purulent penile discharge and a gradually worsening ulcer over the dorsum of his long finger metacarpophalangeal joint (MCPJ; Figure 3). He also reported a truncal rash that started 3 weeks ago. He works as a dishwasher and has been repeatedly picking at the ulcer over this time. In the past week, the area surrounding the wound became swollen, erythematous, and painful. A number of vesicles appeared on the dorsum of the hand as well. Physical examination showed swelling and erythema surrounding the long finger MCPJ. There was an ulcer with hypertrophic scarring over the MCPJ dorsum, with mild tenderness to palpation. There was no pain with movement. He had normal strength and sensation throughout. Review of systems was largely negative except for his penile discharge and hand pain.

#### Diagnosis

Hand x-ray showed some soft tissue swelling with no signs of osteomyelitis. Labs demonstrated a CD4 count of 319, viral load 156 000. An excision of the lesion was performed at bedside and sent for pathology, stain, and cultures. Urethral swab resulted positive for gonorrhea, but negative for *Chlamydia trachomatis*. Blood cultures were negative. After the patient developed vesicular lesions on the face and arms, swabs of vesicular fluid were sent for PCR NAAT testing, and a diagnosis of mpox was confirmed.

#### Treatment

Upon presentation, the patient was immediately restarted on his antiretroviral medications (bictegravir/emtricitabine/tenofovir alafenamide) and ceftriaxone for suspected disseminated gonorrhea. As discussed, the patient’s gonorrhea was not disseminated, and thus 1 dose was adequate to successfully treat this. Vancomycin was added to cover possible superimposed bacterial infection of his unroofed ulcers. This was discontinued after cultures of the lesion were negative on gram stain and culture. He continued his antiretroviral therapy and treated his hand pruritis and swelling with elevation and warm soapy soaks 6 times daily.

#### Disease course/outcome

After treatment of his HIV, the patient’s vesicular lesions improved on hospital day 4, and the patient was discharged home to quarantine. Follow-up visit 23 days after initial presentation confirmed the resolution of all skin changes and no permanent defects. The patient returned to work without complaints.

## Discussion

Patients presenting with hand lesions and a history of uncontrolled HIV should create a high index of suspicion for mpox infection. These patients are usually not surgical candidates as resolution of their lesions is best achieved with medical management such as antiretroviral therapy to control their viral load and immunosuppressed state. We recommend admission for medical management, wound care, wound samples, an infectious disease consult, and prompt contact precautions for patients with suspected mpox. Confirmation of mpox infection is based on NAAT using PCR. Skin lesion surfaces or exudate should be swabbed and roofs from more than 1 lesion or lesion crusts should sent for testing if possible.^8^ If the patient has a history of HIV, or HIV is suspected, an HIV panel should be sent for CD4 counts and viral loads.

### What to Look Out for

**Figure 4.**
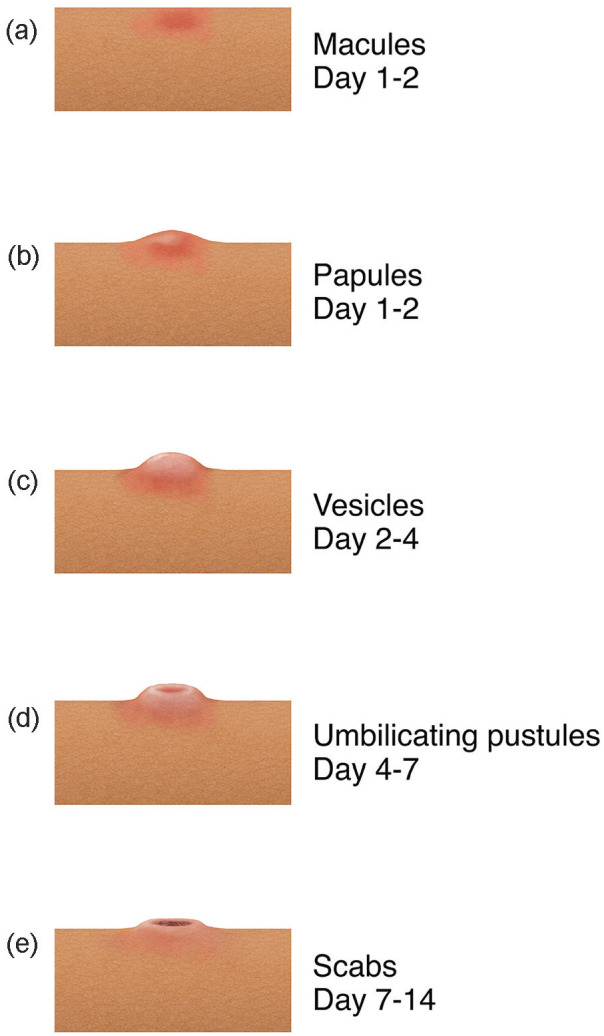
Typical progression of mpox lesions over time from (a) macules (days 1 and 2) to (b) papules (days 1 and 2) to (c) vesicles (days 2-4) to (d) umbilicating pustules (days 4-7) to (e) scabs (days 7-14).

For patient A, mpox suspicion was high upon presentation due to his whole-body rash. However, in the case of patient B, his hand lesion was the first potential hint to mpox, which was not confirmed until 5 days after initial presentation (after discharge). Key characteristics of the mpox rash to watch out for include isolated or disseminated lesions (Figure 4) that are well-circumscribed, firm or rubbery, umbilicated, and painful with progression to pruritis during the healing phase.^9^ These lesions may first present on the hands before becoming apparent elsewhere on the patient.

### Personal Protective Equipment and Exposure Considerations

Mpox is spread through contact with saliva, upper respiratory secretions, and areas around the anus, rectum, or vagina. Risk is considered low for transmission of mpox by touching objects, fabrics, or surfaces that may have been used by someone with mpox and not disinfected.^10^ The correct use of personal protective equipment is highly effective at preventing transmission of mpox. The CDC recommends a gown, gloves, eye protection, and an NIOSH (National Institute of Occupational Safety and Health)–approved particulate respirator equipped with N95 filters or higher when interacting with mpox patients. In the case of unprotected contact between a health care worker’s intact skin and possibly contaminated bodily fluids or soiled materials from an mpox patient (intermediate risk), monitoring and postexposure prophylaxis (PEP) are recommended on an individual basis to decide whether the benefits of PEP outweigh the risks of transmission or severe disease. In higher risk exposures, such as splashes of patient saliva into the eyes or mouth of a worker, monitoring and PEP is recommended. Depending on severity of exposure, exposed asymptomatic health care workers do not need to be excluded from work but should be monitored for symptoms for 21 days following exposure.^11^ Any concerns for possible exposure should be discussed with hospital infection control for next appropriate steps.

### Wound Care

Compromised skin areas should be managed with soap and water or diluted water povidone-iodine solution, moisturized dressings, and topical antibiotics (eg, silver sulfadiazine). Superinfection of the skin is possible and managed with antibiotics, incision and drainage, or negative pressure wound therapy.^12^ If lesions are extensive enough to limit range of motion, such as in the case of patient A, hand therapy may also be of benefit.

## Conclusions

It is important to emphasize that these are 2 very different cases with different outcomes. Patient A’s case demonstrates the potential severity of mpox and its progression. Patient B is an example of how mpox may go initially unnoticed if clinicians do not have high suspicion and a keen eye for the disease as the patient was discharged from the hospital before confirmation of mpox was even obtained. While the daily cases of mpox in the United States have begun to plateau, we advise clinicians to continue to be aware of mpox and how it may first present as a hand consult in the ED. We advise hand doctors to have a high degree of suspicion for mpox when patients with immunodeficiency such as HIV present to the ED with new hand lesions.
